# Cognitive Assessments Used in Occupational Therapy Practice: A Global Perspective

**DOI:** 10.1155/2020/8914372

**Published:** 2020-08-12

**Authors:** Fahad S. Manee, Mohammed Shaban Nadar, Naser M. Alotaibi, Mehdi Rassafiani

**Affiliations:** Occupational Therapy Department, Faculty of Allied Health Sciences, Kuwait University, Kuwait

## Abstract

This exploratory study was aimed at evaluating the current status of global occupational therapy practice on the use of assessments for clients with cognitive impairments and providing recommendations for ongoing evidence. We targeted international occupational therapy clinicians working with clients experiencing neurocognitive impairments. 323 occupational therapists from a wide range of clinical practice areas participated in the study. A large number of therapists used noncognitive specific assessments with a focus on functional approaches. The most commonly used standardized assessments were the COPM (56.7%), followed by MMSE (54.2%) and MoCA (45.5%), while the nonstandardized assessments were clinical observation (38.4%) and generic ADL assessment (34.1%). The use of main assessments was significantly different across world regions (*p* < 0.05), as were the reasons for choosing them (*p* < 0.05). The occupational therapists' use of assessment tools with clients suffering from neurocognitive impairments is inconsistent across the globe. The identification of international best practices for selecting and implementing proper outcome measures is warranted. It is essential to promote the development of an occupational therapy initiative to support the use of appropriate assessments at the international levels to facilitate consistent best practice.

## 1. Introduction

Cognition refers to the conscious and unconscious information processing functions that are carried out by the brain [1]. Cognitive abilities include elementary skills such as arousal, alertness, and orientation, as well as higher cognitive skills such as insight, judgment, and problem solving. The evaluation of the cognitive abilities of individuals with cognitive deficits is vital to the therapeutic process [2]. A proper assessment will determine the individual's ability to live independently, resume productive life roles, and form new habits and routines [3].

Occupational therapists are constantly urged to implement standardized outcome measures in practice [4, 5]. Nonetheless, some therapists remain reliant on nonstandardized assessments, such as informal subjective observations [6]. Burns and Neville (2016) reported that 96% of occupational therapists working in home healthcare treating adult stroke clients used nonstandardized assessments. Therapists working in pediatric settings reported the use of standardized assessments more frequently than practitioners working in adult settings [7]. This inconsistent use of standardized assessments appears prevalent around the globe. In Brazil, for example, the majority of outcome measures have not been validated for use in clinical practice [8], and only a few cognitive assessments have been cross-culturally adapted for use in the Brazilian culture and language.

In accordance with the International Classification of Functioning, Disability and Health [9], occupational therapists working in cognitive rehabilitation can evaluate an individual's cognitive performance at two main levels. The first level, “body structure and function,” considers cognition in terms of the performance of cognitive components such as memory, attention, information processing, and executive functions. This is also known as the “bottom-up” approach [10]. Some examples of assessment tools at this level include the general cognitive measures of the Mini-Mental Status Examination (MMSE) and the Montreal Cognitive Assessment (MoCA). Moreover, there are assessment tools for specific cognitive domains such as memory (e.g., Rivermead Behavioral Memory), processing speed (e.g., Trail Making Test), and executive functioning (e.g., Wisconsin Card Sorting Test).

The second level, “activity and participation,” considers how cognition enables an individual to successfully engage in daily occupations such as basic and instrumental activities of daily living (ADL/IADL). This is known as the “top-down” approach [10] and refers to the therapist's observation of a client's performance of everyday tasks to ascertain cognitive abilities. This level includes assessment tools such as interviews with the client and relevant others, as well as occupational performance-based assessments (e.g., Functional Independence Measure (FIM), Kitchen Task Assessment, Performance Assessment of Self-Care Skills (PASS), and Canadian Occupational Performance Measure (COPM)).

Occupational performance-based assessments are defined as those standardized or nonstandardized methods that involve a therapist observing an individual's performance at daily activities (e.g., FIM and PASS) [11] or a client's self-perception of his/her occupational performance over time (e.g., COPM). Skilled observations are generally a nonstandardized method of evaluating real-world performance in a naturalistic context [4]. A client's active participation is becoming a key component in healthcare systems because it aligns with the client's preferences, needs, and values.

To identify the current cognitive assessment tools used in occupational therapy practice, several researchers surveyed clinicians working in various clinical settings in developed countries, including Canada [11], USA [12], Australia [13], Sweden, and Japan [14]. Many respondents reported the use of standardized assessment approaches because they consider them to be more “formal” assessments of cognition to help identify the client's deficits. Some of the most commonly used measures were MMSE, FIM, and the Barthel Index (BI). While occupational therapists play an important role in cognitive rehabilitation by completing occupational performance-based assessments, the results indicate that therapists utilized assessments focused on the “body structure and function” more than using assessments focused on “activity and participation” level [13]. Occupational therapists often find it challenging to integrate occupational-based assessments in daily practice. This has been noted specifically by occupational therapists in Sweden and Japan who found it difficult to use occupational therapy-specific assessments in daily practice [14]. The challenges commonly cited included the scarcity of time in relation to the volume of work needed to be done, the effort required to administer and score the assessment, and sometimes the physical spaces and home environment simulation required to implement occupational-based assessments [7, 14, 15]. These challenges suggest that the selection of assessments by the occupational therapist might be influenced by the ease of incorporation of the tool in practice rather than the appropriateness of the assessment to the client's individualized case or the soundness of its psychometric properties.

The currently available research that compares the use of cognitive assessments among occupational therapists at a global level is somewhat limited. The aim of this study was to evaluate the current status of global occupational therapy practice on the use of assessments when working with client populations with neurocognitive impairments.

## 2. Materials and Methods

### 2.1. Study Design

To examine the aim of the study, we utilized an exploratory, cross-sectional methodology containing checklists and close-ended questions to gather therapists' perspectives regarding outcome measures. A descriptive survey methodology allows gathering data on respondents' opinions and detailed information pertaining to demographic profiles, while ensuring consistency of questions across all respondents [16]. Ethical approval was obtained from the local university Institutional Review Board and Human Ethics Committee (approval # 160518).

### 2.2. Participants

The study participants were occupational therapists from around the globe attending the World Federation of Occupational Therapists (WFOT) Congress in Cape Town, South Africa, in 2018. The inclusion criterion was that the occupational therapists must be regularly working with clients experiencing neurocognitive impairments for at least two years. We excluded students and novice therapists from participation as the study focus was on experienced clinicians.

### 2.3. Instrument Development and Validation

We compiled a comprehensive list of cognitive assessments used in occupational therapy practice around the world by conducting a thorough search of health-related databases, occupational therapy books, and occupational therapy journals. We searched the PubMed and CINAHL electronic bibliographic databases from the years 1995 to 2018. The search strategies included variations and a combination of the terms “cognition, occupational therapy, assessment and outcome measures”. Additionally, we scanned the reference lists of identified studies and reviews. Since nonstandardized assessments are frequently used by occupational therapists [11], we included both standardized and nonstandardized choices in our list of assessments.

To validate our list, we presented it to a group of experts with different occupational therapy backgrounds, who were instructed to review and modify the list according to the objective of the study. The expert panel consisted of four Ph.D. holders and experienced occupational therapists from Iran, USA, India, and Kuwait, with an average of 23.7 years of working experience (SD = 4.2) in diverse clinical backgrounds including neurocognitive rehabilitation, mental health, and pediatrics. After several meetings, the expert panel agreed on a final list of 98 assessments to be included in the study. To maximize utility, the assessments were listed in alphabetical order and the main questions were formulated to be close ended.

### 2.4. Structure and Format

The final version of the survey consisted of three main sections.  Section 1 targeted the demographics of the study participants, including the age, gender, country of residence, professional degree, years of clinical experience, areas of practice, and settings of practice.

Section 2 included an alphabetical list of 98 assessments (both occupation-based and skill-based) that can be used with clients experiencing neurocognitive impairments. The participants were instructed to choose all the ones they routinely used (Table 1). Due to the wide variety of professional backgrounds among the potential respondents, we also provided an additional space where nonlisted assessments could be added if needed.

Section 3 was related to the factors affecting assessment choices. The participants were allowed to select multiple factors that contributed to their assessment choices. The list of reasons provided in the survey was based on the occupational therapy literature [11, 12] as well as the authors and expert panel feedback. The reasons were meant to reflect the main rationale for choosing the assessment method in all practice areas. They included availability in the work setting, established psychometric properties, client centeredness, ease of administration, and pertinence to a specific frame of reference.

### 2.5. Statistical Analyses

We used descriptive statistics to calculate the means, standard deviations, frequencies, and percentages. Nonparametric chi-square tests were used to compare the demographic data with assessment preferences and the prevalent rationales for choosing them, as well as to test for geographical differences in the use of an assessment tool. We employed Kruskal-Wallis ANOVA tests to compare between two or more independent categories. *p* values < 0.05 (2-tailed) were considered significant. Statistical analyses were performed using the SPSS software (version 25.0; SPSS Inc., Chicago, IL) for Windows.

## 3. Results

### 3.1. Demographic Characteristics of Participants

A total of 323 participants completed the survey. The demographic characteristics of the respondents (Table 2) showed that the majority were female (86.4%), and the largest age group was 40+ years (40.2%). Almost half of the respondents had more than 10 years of working experience (46.4%).

### 3.2. Common Assessments Used by Participants

The study listed 98 standardized and nonstandardized assessment tools. The participants indicated which assessment(s) they typically used in their clinical practice.  Table 3 shows the ten most popular tools as selected by the therapists. The COPM was the most popular choice (56.7%) followed by MMSE (54.2%) and MoCA (45.5%). There were also two nonstandardized assessment tools among the ten most popular, namely, clinical observation (38.4%) and general ADL assessment (34.1%). There were very few responses in the extra space provided to allow additional assessments, and the qualitative data did not significantly add to the findings of the study. We will therefore not report the qualitative findings.

### 3.3. Geographical Differences in Assessment Tool Choice

Chi-square analyses revealed that the use of COPM, MoCA, FIM, and the Glasgow Coma Scale varied significantly (*p* < 0.05) across the world regions. Furthermore, the frequency of choosing MMSE (*p* = 0.039), MoCA (*p* = 0.017), clinical observation (nonstandardized) (*p* = 0.011), and the Clock Drawing Test (*p* = 0.045) varied significantly with the level of the therapist's years of professional experience, where more experienced therapists selected these assessments less frequently.

Therapists with higher education degrees (i.e., above a bachelor's degree) employed the MMSE (*p* = 0.049) and nonstandardized ADL assessments (*p* ≤ 0.001) less often, and the FIM more often (*p* = 0.031), than therapists with BSc degrees. The other assessment tools did not show any statistically significant difference regarding the therapist's educational degree. We also did not find any significant differences with respect to the therapist's gender.

### 3.4. Reasons for Using an Assessment

The most common reasons for using an assessment was “Available where I work” (79.6%) and “I am familiar with the assessment” (65.9%). The full list of reasons is shown in Table 4. Chi-square analyses showed significant differences in reasons for using the assessment based on the geographical regions of the world.  Table 5 shows more details on the reasons for selecting an assessment tool based on geographical regions.

When comparing reasons for using an assessment based on years of experience, the Kruskal-Wallis ANOVA found a statistically significant difference between therapists with more than 10 years of experience and those with less than 10 years of experience. More specifically, experienced therapists (10+ years) relied more on assessments that follow a specific occupational therapy frame of reference (*p* = 0.021), did not rely on colleagues' preferences (*p* ≤ 0.001), preferred valid and reliable assessments (*p* = 0.007), were not biased towards assessments addressed in their OT curricula (*p* ≤ 0.001), and did not rely on assessments they had learned postuniversity training (*p* = 0.007).

## 4. Discussion

The main aim of this study was to evaluate the current status of global occupational therapy practice on the use of assessments with clients experiencing neurocognitive impairments. The Canadian Occupational Performance Measure (COPM) was the most widely used assessment, where 56.7% of our respondents reported using the COPM. The wide use of this measure is consistent with earlier studies specific to cognitive impairments [11] as well as other general occupational therapy assessments [12]. The COPM is not a dedicated cognitive assessment, but rather a generic client-centered measure that applies to most diagnoses. This high usability of the COPM with cognitive impairment diagnoses emphasizes the importance of active participation of clients in formulating their own goals to international occupational therapy practice. A scoping review concluded that the use of the COPM can enhance client-centered practice by improving awareness of the client's goals for the future, thereby reinforcing a partnership with a collaborative goal setting [17].

Despite its overall prevalence, therapists working in the African continent used the COPM significantly less than their colleagues from other regions of the world (38.8% vs. 67.1%, respectively). Overall, occupational therapists in Africa tended to rely more on skill-based rather than occupation-based assessments. In a study that explored the challenges faced by occupational therapists in South Africa when planning to implement occupation-based assessments and interventions, the barriers identified included lack of availability of assessments, unfamiliarity with the occupation-based assessment approach, reimbursement problems, and therapist preferences [18].

Given the wide adoption of COPM, the question arises of whether this standardized assessment can be sufficient to provide a comprehensive understanding of a client's condition. Findings from randomized control trials revealed that most occupational therapists used other standardized assessments in conjunction with COPM to obtain a more comprehensive overview of their clients' needs [19]. This result is further supported by a systematic review that reported the COPM to be more effective when used in conjunction with other assessments such as the Barthel Index (BI) and the Functional Independence Measure (FIM) [20]. Our study found that many of our respondents used the FIM (34.1%) and the BI (31.6%), which is correspondingly consistent with previous observations [12, 13]. The FIM and BI provide measures for the level of assistance required by an individual to perform basic daily life activities and are not dedicated measures of cognitive skills and abilities. Occupational therapists working with cognitively impaired clients may find the FIM to be particularly valuable because it includes a cognitive subscale in addition to the self-care information it provides. Of note, occupational therapists from the Asia and Pacific regions used the FIM (53.8%) and BI (44.2%) more frequently than therapists from the remaining regions (31.9% and 29.6%, respectively).

With regard to skill-based cognitive assessments, our respondents used the MMSE (54.2%), MoCA (45.5%), Clock Drawing Test (30.3%), and Glasgow Coma Scale (28.2%) more frequently than any other cognitive assessment tools. A survey by Koh et al. (2008) found a particularly high prevalence of MMSE in Australia, and a different study reported it to be the most frequently mentioned assessment among clinicians in Canada [11]. A high prevalence of MMSE and MoCA assessments was also found in other studies [11, 21]. The aims of the MMSE and MoCA approaches are comparable as both are routine cognitive screening tests rated on a 30-point scale that attempt to detect mild cognitive impairment. However, given the documented advantages of the MoCA, the abundant use of the MMSE by global healthcare professionals is somewhat surprising. Of note, the MoCA has been found to have higher sensitivity than the MMSE in detecting cognitive deficits, especially in the domains of visuospatial/executive function, attention, and recall, in addition to its superiority in detecting cognitive deficits in patients with transient ischemic attack and stroke [22]. The MoCA is also reported to be a better measure of cognitive status for some diagnoses as it lacks ceiling and floor effects [23]. One reason for the abundant use of the MMSE may be related to the dominance of the medical model in many service settings. Another factor may be due to the potential gap between the research-based scientific evidence and actual clinical practice.

The results of this study showed that therapists with higher education degrees (i.e., above a bachelor's degree) employed the MMSE and nonstandardized ADL assessments less often, and the FIM more often, than therapists with BSc degrees. There are no differences between these groups in other assessment tools. Therapists with higher education degrees might have higher knowledge and skills about research and evidence-based practice compared to undergraduate therapists. This may be a reason that therapists with higher degrees choose FIM, which is more valid and reliable. However, more research is required to target educational backgrounds and find if there are differences between these two groups in using other types of assessment tools.

The most cited reason by our respondents for using an assessment was its “availability at work” (79.6%), which was consistent across all world regions. A previous study of occupational therapists in the United States found the same dominant reason for choosing an assessment [12]. Such practice, which is based on availability rather than applicability, can directly affect the occupational therapy intervention process. For this reason, a particular focus should be placed on managers and decision makers to ensure the availability of a wide range of suitable assessment tools in their clinics.

The occupational therapy literature emphasizes the need to incorporate standardized assessments with psychometrically sound properties in clinical practice [24]. Accordingly, the majority of respondents in this study acknowledged psychometric properties to be a main factor for selecting an assessment. Nevertheless, two of the ten most used assessments identified in this study were not standardized. Nonstandardized assessments can be useful, but they may also be compromised as their results may not be reliable or valid in the first place. Furthermore, the use of nonstandardized assessments in certain professions also carries the risk of not being properly interpreted by the wider healthcare community. Occupational therapists should be continuously encouraged to use standardized assessments to minimize bias and interpretation errors.

The time-consuming nature of some standardized outcome measures has been previously identified as an obstacle in clinical settings [25]. This was also evident in this study where respondents identified time (“quickly administered”) and clinical utility of the assessment (“easily interpreted”) as major factors for selecting an assessment. Only therapists from Europe did not widely consider the criterion of clinical utility as an important reason for selecting an assessment (28% in Europe vs. 63.3% in the rest of the world). While assessments that are faster to administer may be more efficient and practical in a clinical setting and therefore more readily used, assessments that are selected based on the time factor alone may lack the necessary comprehensiveness or sensitivity to capture the desired outcomes.

A limitation of this study is the relatively small sample size from which a conclusion about geographical variations in the use of assessment tools can be drawn. Another limitation is the convenient sampling methods we implemented in this study. This makes the sample nonrepresentative and may limit the generalizability of the findings in terms of accurately reflecting the prevalence of use in different countries. Given the nature of the WFOT World Congress event, it is likely that the attendees were academicians and more resourced therapists, which may make our sample biased. Nevertheless, this study provides an initial consensus over the prevalence of different types of assessment tools used in cognitive clinical practices across the globe. The findings can be useful to occupational therapy clinicians, educators, researchers, and managers.

In order to support best practice, entry-level education curricula for occupational therapy should emphasize the importance of using occupation-based and standardized assessments to maintain the philosophical underpinning of the profession. The integration of standardized assessments supports the accurate evaluation process and, by extension, emphasizes desired clinical reasoning, planning, and interventions. Since not all assessments, and particularly cognitive assessments, are relevant or practical when used in countries and contexts other than the ones in which they were originally developed [8, 26], international use of assessments developed in other cultures requires careful consideration of the cross-cultural adaptation process to ensure the appropriate use of these assessments, while incorporating linguistic, cultural, and contextual factors. Therefore, in addition to standardization, cultural consideration and awareness should be an integral part of international occupational therapy curricula [27]. International educators are encouraged to teach students the assessments that are not only standardized but also culturally relevant and applicable in their own context.

## 5. Conclusions

The use of valid standardized assessments that match the need of the client is essential for successful and competent therapeutic intervention. In this study, we identified the most commonly used cognitive assessment tools for different geographical regions of the world and the dominant reasons for their use. There is no global consensus on the best assessment tools to use for patients with cognitive impairments. Therefore, it is important to promote the development of an international occupational therapy initiative that supports the use of assessments at both national and international levels. Such an initiative should mainly be geared towards recommending the use of the most appropriate assessments that adequately reflect the philosophy and values of occupational therapy to facilitate the best possible client outcomes. Due to the globalization of occupational therapy practice, it is important to indicate the essence of using assessments that are culturally sensitive and reflective of the targeted population's own culture and beliefs. This will ensure the use of culturally valid and competent assessments.

## 6. Implications for Occupational Therapy Practice

Occupational therapists should entertain the opportunity of periodic professional development on emerging standardized outcome measures pertaining to their clinical settings. Given our findings, we propose the following:

(i) Skill-based cognitive assessments need to be implemented in conjunction with occupation-based assessments to reflect the core principles of occupational therapy practice
(ii) Managers and decision makers should ensure the availability of a wide range of suitable assessment tools in their clinics
(iii) International “assessments in occupational therapy initiative” that is guided by a well-established eminent organization should be developed to
  - foster international collaborations between professional global occupational therapy entities to provide systematic recommendations and guidelines for the use of assessments
  - critically appraise potential international measures and explore ecological validity
  - establish an international occupational therapy assessment database for different areas of practice

## Figures and Tables

**Table 1:** The list of cognitive assessments used in the study.

Name of Assessment		Name of Assessment	
Ability to navigate (non-standardized)	□	Dementia Rating Scale (DRS)	□
Affective Test of Prosody (ATP)	□	Digit Backward Test	□
Allen Cognitive Levels (ACL)	□	Digit Forward Test	□
Assessment of Communication and Interaction Skills	□	Disability Assessment for Dementia (DAD)	□
Assessment of Living Skills	□	Empirical Behavioral Pathology in Alzheimer's Disease Rating Scale (E-Behave-AD)	□
Assessment of Living Skills and Resources (ALSAR)	□	Executive Interview (EXIT)	□
Assessment of Motor and Process Skills (AMPS)	□	Frontal Assessment Battery (FAB)	□
A Quick Test	□	Functional Autonomy Measurement System (SMAF)	□
Barthel ADL Assessment (modified)	□	Functional Independence Measure (FIM)	□
Barthel Index	□	Functional Performance Measure	□
Bay Area Functional Performance Evaluation (BAFPE)	□	General ADL (non-standardized)	□
Bedford Alzheimer Nursing Severity Scale; for the severe demented (BANS)	□	Glasgow Coma Scale (GCS)	□
Canadian Occupational Performance Measure (COPM)	□	Hierarchic Dementia Scale	□
Chessington OT Neurological Assessment Battery (COTNAB)	□	Home (non-standardized)	□
Client-Oriented Role Evaluation	□	Home Environment Assessment Protocol	□
Clinical Observation (non-standardized)	□	Home Falls and Accidents Screening Tool	□
Clock Drawing Test & Clock Test	□	Hopkins Verbal Learning Test (HVLT)	□
Cognitive Adaptive Skills Evaluation	□	Kitchen task (non-standardized)	□
Cognitive Assessment of the Elderly (CASE/Pecpa-2r)	□	Kitchen Task Assessment (KTA)	□
Cognitive Assessment of Minnesota (CAM)	□	Klein-Bell ADL Test	□
Cognitive Assessment Screening Test (CAST)	□	Kohlman Evaluation of Living Skills (KELS)	□
Cognitive Performance Test (CPT)	□	Leisure Satisfactory Questionnaire	□
Cognitive Competency Test (CCT)	□	Leisure Diagnostic Battery	□
Cognitive Mode Questionnaire (CMQ)	□	Limiting Long Standing Illness screen (LLSI)	□
Colored ball sort (non-standardized)	□	Line Bisection Test	□
Comprehensive OT Evaluation (COTE)	□	Loewenstein O.T. Cognitive Assessment (LOTCA)	□
Contextual Memory Test (CMT)	□	Loewenstein O.T. Cognitive Geriatric Assessment (LOTCA-G)	□
D2 test of Attention	□	Middlesex Elderly Assessment of Mental Status (MEAMS)	□
Mini Mental Status Exam (MMSE)/Folstein	□	Trail Making Test (TMT)	□
Model of Human Occupation Screening (MOHOST)	□	Test of Visual Perceptual Skills (Non-Motor)-Revised (TVPS(n-m)R)	□
Montreal Cognitive Assessment (MoCA)	□	Toglia Category Assessment	□
Motor-Free Visual Perceptual Test (MVPT)	□	Visual Field Tests: Bell's Scanning Test, Useful Field of Vision Test	□
Modified Mini Mental Status Exam (3MS)	□	Volitional Questionnaire	□
Neurobehavioral Cognitive Status Examination (NCSE/Cognistat)	□	Weschler Adult Intelligence Scale-III (WAIS-III)	□
Occupational History	□	Wisconsin Card Sorting Test version 4 (WCST-CV4)	□
Occupational Role History	□	Woodcock Johnson Test of Cognitive Ability	□
Occupational Self-Assessment	□	Word list (non-standardized)	□
Ontario Society of O.T. Perceptual Assessment	□	Work Environment Impact Scale	□
Occupational Questionnaire	□	Worker Role Inventory Worker Role Inventory	□
Orientation Test for Aphasics	□	Severe Impairment Battery (SIB)	□
Other IADL tasks (e.g. medication mgmt., financial tasks) (non-standardized)	□	Sorting shapes (non-standardized)	□
Perceive Recall Plan Perform (PRPP)	□	Stroke Unit Mental Status Exam (SUMSE)	□
Performance Assessment of Self-Care Skills (PASS)	□	Stroop test Stroop test Stroop test Stroop test	□
Rancho Los Amigos (RLA)	□	Structured Observational Test of Function (SOTOF)	□
Rivermead Behavioral Memory Test (RMBT)	□	Tests designed for acquired brain injury (SCATBI,NRS)	□
Role Check List	□	Test of Everyday Attention (TEA)	□
Ross Information Processing Assessment – Geriatric (RIPA-G)	□	Test for Severe Impairment (TSI)	□
Routine Task Inventory Routine Task Inventory	□	Test of Visual Motor Skills – Revised (TVMS-R)	□
Safety Assessment of Function and the Environment for Rehabilitation (SAFER)	□	OTHERS: Please list below) ……………………………………………………..	□
Safety and Functional ADL Evaluation (SAFE)	□	……………………………………………………	□

**Table 2:** Demographic characteristics of the participants.

Participants' characteristics	Frequency	Percent
Age (year)		
21-30	102	31.6
31-40	88	27.2
>40	130	40.2
Years of experience		
Up to 5	98	30.3
5-10	61	18.9
More than 10	150	46.4
Region of residence		
N. & S. America	78	24.1
Europe	75	23.2
Asia and Pacific	52	16.1
Africa	116	35.9
Area of practice		
Pediatrics	105	32.5
Neurorehabilitation	134	41.5
Community-based	60	18.6
Geriatrics	60	18.6
Mental health	71	22.0
School-based	36	11.1

Note: numbers may not add up to the total due to missing responses.

**Table 3:** Top ten assessments used by the participants.

	Tool/assessment	Frequency	Percent
1	Canadian Occupational Performance Measure (COPM)	183	56.7
2	Mini-Mental Status Exam (MMSE)	175	54.2
3	Montreal Cognitive Assessment (MoCA)	147	45.5
4	Clinical observation (nonstandardized)	124	38.4
5	Barthel ADL assessment (modified)	121	37.5
6	Functional Independence Measure (FIM)	110	34.1
7	General ADL assessment (nonstandardized)	110	34.1
8	Barthel Index (BI)	102	31.6
9	Clock Drawing Test	98	30.3
10	Glasgow Coma Scale	91	28.2

Note: some participants used more than one tool/assessment in their practice.

**Table 4:** The reasons for using assessments (ordered from the top to the lowest reason).

	Reasons for choosing assessment	Frequency	Percent
1	Available where I work	257	79.6
2	I am familiar with the assessment	213	65.9
3	Client-centered	197	61.0
4	Has known reliability and validity	184	57.0
5	Quick to administer	175	54.2
6	Easy to interpret	155	48.0
7	I learned it during my professional training	138	42.7
8	Follows a specific OT frame of reference (FOR)	122	37.8
9	Colleagues recommend its use in practice	110	34.1
10	Taught in the OT educational curriculum	105	32.5
11	Found it through a literature search	104	32.2
12	Developed by an occupational therapist	103	31.9
13	Described in a professional textbook/journal	56	17.3
14	I heard about it at a conference/seminar	40	12.4
15	Satisfies insurance company	29	9.0
16	Follows a specific FOR outside OT profession	26	8.0
17	Newly developed	16	5.0

Note: the participants may indicate more than one reason.

**Table 5:** The reasons for using the assessment tool by the geographical region.

Rationale for using assessment		N. & S. America		Europe		Asia and Pacific		Africa		*p* value
		No	Yes	No	Yes	No	Yes	No	Yes	
Available where I work	Frequency	16	61	24	51	8	44	16	100	0.016^∗^
	Percent	20.8%	79.2%	32.0%	68.0%	15.4%	84.6%	13.8%	86.2%	
Client-centered	Frequency	31	46	19	56	17	35	57	59	0.008^∗^
	Percent	40.3%	59.7%	25.3%	74.7%	32.7%	67.3%	49.1%	50.9%	
Has established reliability and validity	Frequency	28	49	26	49	22	30	62	54	0.034^∗^
	Percent	36.4%	63.6%	34.7%	65.3%	42.3%	57.7%	53.4%	46.6%	
Quick and easy to administer and interpret	Frequency	29	48	54	21	17	35	46	70	≤0.001^∗^
	Percent	37.7%	62.3%	72.0%	28.0%	32.7%	67.3%	39.7%	60.3%	

^∗^The distribution is significantly different between the world geographical regions.

## Data Availability

The data used to support the findings of this study are available from the corresponding author upon reasonable request.
